# Molecular phylogeny of recognised Thai *Landouria* species (Gastropoda, Camaenidae), with descriptions of two new species

**DOI:** 10.3897/zookeys.1278.172545

**Published:** 2026-04-28

**Authors:** Benchawan Nahok, Utain Chanlabut, Kitti Tanmuangpak

**Affiliations:** 1 Program of General Science, Faculty of Education and Human Development, Chaiyaphum Rajabhat University, Muang District, Chaiyaphum, 36000, Thailand Program of General Science, Faculty of Education and Human Development, Chaiyaphum Rajabhat University Chaiyaphum Thailand; 2 Program of Biology, Department of Science, Faculty of Science and Technology, Loei Rajabhat University, Muang District, Loei Province, 42000, Thailand Program of Biology, Department of Science, Faculty of Science and Technology, Loei Rajabhat University Loei Thailand

**Keywords:** 16S rRNA, Camaenidae, COI, genitalia, integrative systematics, phylogeny, taxonomy, terrestrial snail, Thailand

## Abstract

The molecular phylogeny and morphological–anatomical characteristics of the terrestrial snail genus *Landouria* Godwin-Austen, 1918 from Thailand are investigated, and we describe two new species. Phylogenetic analysis of 11 recognised Thai *Landouria* species reveal that these species are phylogenetically well separated from each other by mtDNA phylogeny and COI sequence divergences of 0.048–0.192. *Landouria tumpeesuwanorum***sp. nov**. is described from a limestone hill in Nong Bua Lamphu Province, northeastern Thailand; it is characterised by its angulated whorls, a strongly keeled shell, and small flagellum with curved ends. The second new species, *Landouria flagellolonga***sp. nov**. is described from a limestone hill in Sa Kaeo Province, eastern Thailand. It has a conical-lenticular, sharply keeled shell, and a very long, slender flagellum.

## Introduction

Species of the terrestrial snail genus *Landouria* Godwin-Austen, 1918 (Gastropoda, Camaenidae) from Thailand were previously classified within the genus *Aegista* Albers, 1850 (Bradybaenidae) based solely on shell morphology. The taxonomic history of Thai *Landouria* began with a nominal species described by Solem (1966), followed by six nominal species by Panha (1996) and five nominal species by Hemmen and Hemmen (2001) (see Tumpeesuwan and Tumpeesuwan 2019: Table 2). Subsequently, Schileyko (2004) established a new genus, *Thaitropis*, which shared conchological similarities with *Landouria* but was distinguished by its reproductive anatomy, specifically a sharp narrowing between the epiphallus and penis; however, Nurinsiyah et al. (2019) later synonymized *Thaitropis* with *Landouria*. Although Boonngam et al. (2008) reported *L. winteriana* (Pfeiffer, 1842) and *L. smiroensis* (van Benthem Jutting, 1950) from Thailand, these records were based only on shell characters. Subsequent studies by Köhler et al. (2018) and Nurinsiyah et al. (2019) concluded that the two species are restricted to the Indonesian Archipelago, suggesting that the specimens from Thailand may have been confused with an undescribed species, as only shell morphology had been reported for those records.

A significant advance was made when Tumpeesuwan and Tumpeesuwan (2019) provided the first verified anatomical and radula data for a species of *Landouria* from Thailand, supplementing the existing conchological-based taxonomy. Subsequent molecular phylogenetic work by Nahok et al. (2021) confirmed that *Landouria* from Thailand is distinct from the Indonesian Archipelago group and described six additional species. Further studies by Nahok et al. (2024) described two additional species based on reproductive anatomy, radula morphology, and shell characters. A more recent study, Páll-Gergely et al. (2025), described one additional species of *Landouria* from southern Thailand based solely on unique shell characteristics.

As a result of these recent efforts, 10 valid species of *Landouria* are currently recognized from Thailand, most of which are endemic to limestone hills in northeastern Thailand: *L. strobiloides* C. Tumpeesuwan & S. Tumpeesuwan, 2019, *L. circinata* Nahok et al., 2021, *L. tuberculata* Nahok et al., 2021, *L. trochomorphoides* Nahok et al., 2021, *L. chloritoides* Nahok et al., 2021, *L. elegans* Nahok et al., 2021, *L. diplogramma* (Möllendorff, 1902) and *L. monodon* Nahok & C. Tumpeesuwan, 2024. A single species is known from western Thailand, *L. bella* Nahok & S. Tumpeesuwan, 2024, and one from southern Thailand, *L. canalifera* Páll-Gergely, S. Tumpeesuwan & C. Tumpeesuwan, 2025.

Despite this progress, the diversity of the genus in the region remains incompletely documented. Here, this study provides an updated molecular phylogeny for the recognised Thai *Landouria* species. Using an integrative approach that combines this phylogenetic framework with morphological and anatomical data, we describe two new species.

## Materials and methods

### Snail collection and preparation

**Figure 1.**
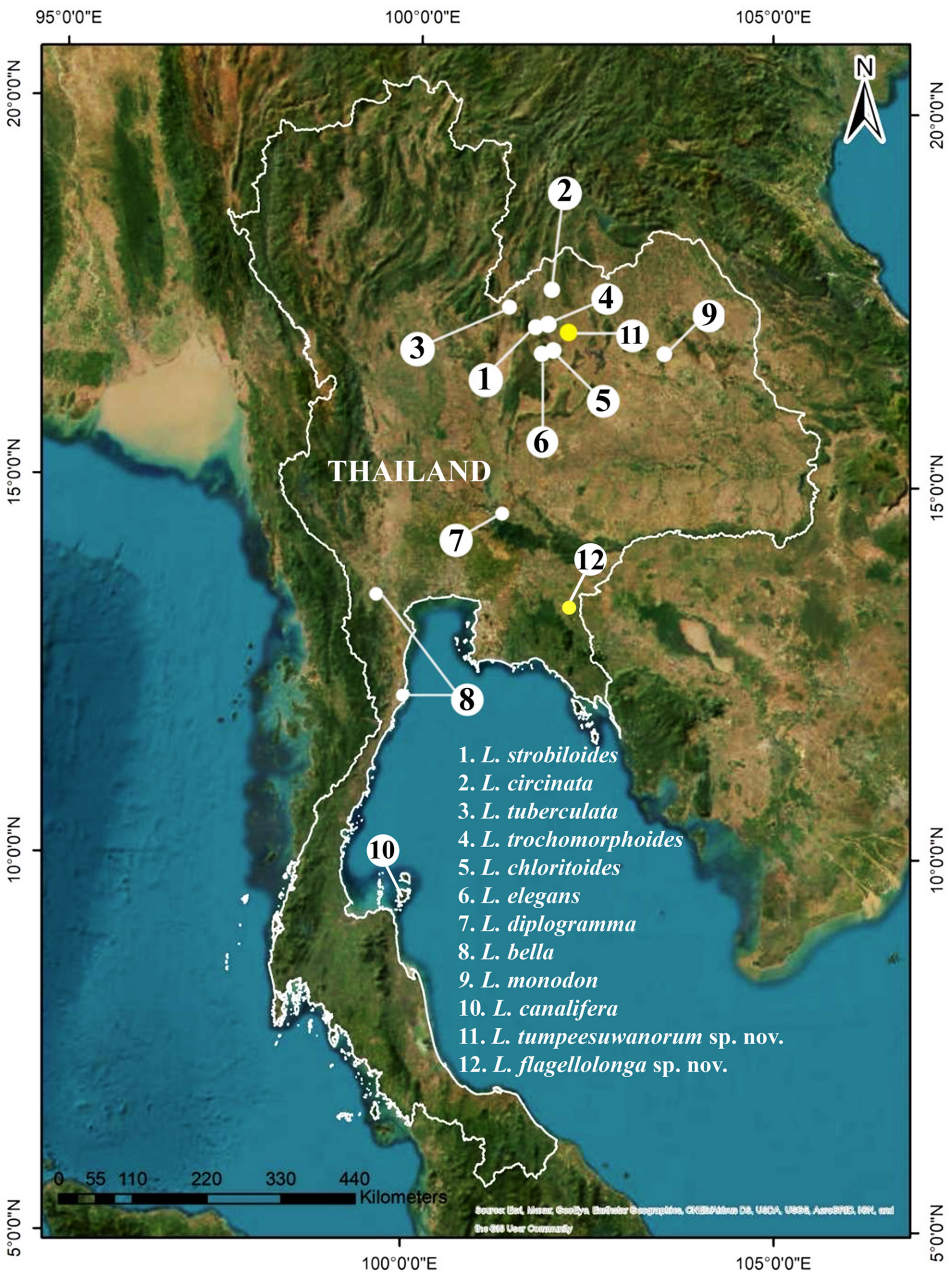
Geographic distribution of verified Thai *Landouria* species and showing the type localities of *L. tumpeesuwanorum* sp. nov. and *L. flagellolonga* sp. nov.

Empty shells and living specimens were collected from two localities: a limestone hill at Pha Sam Yod, Si Bun Rueang District, Nong Bua Lamphu Province, northeastern Thailand, and from Phet Pho Thong Cave, Khlong Hat District, Sa Kaeo Province, eastern Thailand (Fig. 1).

**Table 1.** List of specimens used in the molecular phylogenetic analysis, including locality details, collection catalogue numbers, and GenBank accession numbers. HNHM = Hungarian Natural History Museum; AM = Australian Museum; NHMSU = Natural History Museum, Mahasarakham University; ZCPRU = Zoological Research Collection of Chaiyaphum Rajabhat University; NHLRU = Natural History Museum of Loei Rajabhat University. References: 1 = Köhler et al. (2018); 2 = Tumpeesuwan and Tumpeesuwan (2019); 3 = Nahok et al. (2021); 4 = Nahok et al. (2024); 5 = Hirano et al. (2014); 6 = Hirano et al. (2019); 7 = Hayashi and Chiba (2000).

Taxon	Localities	Type status	Museum No.	Specimen voucher	GenBank accession No.		References
					COI	16S	
*L. omphalostoma*	Yunnan, China		HNHM 98758	PGB—b	MH521049	MH521069	1
*L. rotatoria*	Sumatra, Indonesia		–	PGB—b	MH521051	MH521071	1
*L. winteriana*	Timor-Leste		AM C. 477078	–	MH521065	MH521087	1
*L. montana*	Ramelau Mountains, Timor-Leste		AM C. 477070	FK—2019	MH521055	MH521075	1
*L. timorensis*	Atauro Island, Timor-Leste		AM C. 470207	–	MH521063	MH521078	1
*L. strobiloides*	Suan Hin Pha Ngam, Loei, Thailand	Paratype	NHMSU—00018	NLLESH660	MN449400	MZ435745	2
*L. circinata*	Phu Pha Lom Forest Park, Loei, Thailand	Paratype	NHMSU—00024	NLLEPL660	MN449401	MZ435746	3
*L. tuberculata*	Wat Thepnimit, Loei, Thailand	Paratype	NHMSU—00026	NLLEPR960	MN449402	MZ435747	3
*L. trochomorphoides*	Pha Sawan Cave, Loei, Thailand	Paratype	NHMSU—00028	NLLEPS1060	MN449403	MZ435748	3
*L. chloritoides*	Wat Thep Udom Wanaram, Khon Kaen, Thailand	Paratype	NHMSU—00030	NLKKPNB660	MN449404	MZ435749	3
*L. elegans*	Phu Hua Chang, Khon Kaen, Thailand	Paratype	NHMSU—00033	NLKKHCW561	MN449408	MZ435751	3
*L. diplogramma*	Wat Thep Phithak Punnaram, Nakhon Ratchasima, Thailand		NHMSU—00034	NLKRTP660	MN449411	MZ435752	3
*L. bella*	Khao Bin Cave, Ratchaburi, Thailand	Paratype	ZCPRU—0042	NLRRKB663	PV849288	PP988693	4
*L. monodon*	Phu Po, Kalasin, Thailand	Paratype	ZCPRU—0046	NLKSPP960	MN449413	–	4
*L. tumpeesuwanorum* sp. nov.	Pha Sam Yod, Nong Bua Lamphu, Thailand	Paratype	NHLRU026	NLNBPS1164	PV849287	PP988695	This study
*L. flagellolonga* sp. nov.	Phet Pho Thong Cave, Sa Kaeo, Thailand	Paratype	ZCPRU—0050	NLSKPTR1060	MN449414	–	This study
*Euhadra peliomphala*	Izu Peninsula, Nagaoka, Japan		–	–	AB852700	AF104053	5, 7
*Bradybaena similaris*	Aoba, Sendai, Miyagi, Japan		–	–	AB852697	LC472125	5, 6

All specimens were handled and euthanised following the ethical guidelines of the American Veterinary Medical Association (AVMA 2020), under a protocol approved by the Loei Rajabhat University and Mahasarakham University, Animal Care and Use Protocol Review (No. A 004/2563 and IACUC-MSU-078-068/2025, respectively). The foot muscle tissue of each specimen was snipped, preserved in 95% (v/v) ethanol, and stored at −20 °C for DNA analysis (three specimens per species). The remaining parts were stored in 70% (v/v) ethanol for anatomical study and radula morphology. All materials examined in this study were deposited in the collection of the Natural History Museum, Mahasarakham University (**NHMSU**); the Natural History Museum of Loei Rajabhat University (**NHLRU**), and the Zoological Research Collection of Chaiyaphum Rajabhat University (**ZCPRU**).

Intact adult shells were measured for whorl number and for shell height (**SH**), shell width (**SW**), aperture height (**AH**), and aperture width (**AW**) using digital vernier callipers, and to count the number of whorls. The genitalia of three specimens per species were dissected under a stereomicroscope, and one representative preparation for each was photographed. The radula was extracted from the buccal mass of one specimen per species and examined using a scanning electron microscope (SEM), following the methods of Geiger et al. (2007).

### DNA extraction and amplification

DNA was extracted from small parts of the foot using the GF-1 Nucleic Acid Extraction Kit (Vivantis Technologies Sdn. Bhd, Malaysia) following the manufacturer’s protocol. Sequences of two partial mitochondrial markers (COI and 16S rRNA) were used to construct a phylogenetic tree of the Thai *Landouria* species. The COI was also used to evaluate genetic divergences (*p*-distance) among species. The COI fragment was amplified using the primers L1490 and H2198 (Folmer et al. 1994), while the 16S rRNA fragment was amplified using 16Scs1 (Chiba 1999) and 16S_MN3R (Neiber et al. 2017). PCR reaction conditions followed Nahok et al. (2021). The PCR products were checked using a 1% agarose gel electrophoresis stained with 1× Novel Juice Loading Dye (GenDirex^®^, Taiwan, China). Successful amplifications were sent for NGS-based sequencing at Celamics DNA Sequencing Services (Seoul, Korea) and 1^st^ BASE DNA Sequencing Services (Selangor, Malaysia).

### Phylogenetic analysis

Forward and reverse sequences were checked using Bioedit v. 7.7 (Hall 1999, 2001). Sequences were aligned using the Clustal W algorithm in MEGA X (Kumar et al. 2018). The newly obtained nucleotide sequences in this study were deposited in the GenBank database under accession numbers PV849287, PV849288, MN449413, and MN449414 for COI; and PP988693 and PP988695 for 16S rRNA with sequence lengths of 655 bp and 460 bp, respectively. Additionally, we included GenBank sequences of other *Landouria* from Thailand (Nahok et al. 2021), Timor-Leste, China, and Sumatra, Indonesia (Köhler et al. 2018) in our analyses for genetic distance and phylogenetic analyses, and we used sequences of *Euhadra peliomphala* (Pfeiffer, 1850) and *Bradybaena similaris* (Férussac, 1822) as outgroups following Hirano et al. (2014) and Nurinsiyah et al. (2019). Two missing 16S rRNA sequences were coded as missing data in the concatenated dataset. Collection localities and accession codes for each nominal species are shown in Table 1. Interspecific genetic divergences were calculated with pairwise *p*-distances based on the Kimura 2-parameter model (Kimura 1980) computed using MEGA X (Kumar et al. 2018).

The aligned COI and 16S rRNA sequences were concatenated into a single partitioned dataset using the program EMBOSS seqret (Madeira et al. 2019). The final concatenated alignment from all *Landouria* species was used to construct phylogenetic relationships using the Bayesian inference (BI), maximum likelihood (ML), and neighbour-joining (NJ) methods. The program jModelTest v. 2.1.10 (Posada 2008; Darriba et al. 2012) was used to determine the best-fitting models of DNA substitution using the Akaike Information Criterion (AIC) (Akaike 1974). The best-fit models for each partition were as follows: TPM1uf+I+G (1^st^ codon position of COI), TPM3uf+G (2^nd^ codon position of COI), TIM1+I+G (3^rd^ codon position of COI), and GTR+G (16S rRNA). For the concatenated dataset, GTR+I+G was selected as the best-fit model.

The unpartitioned NJ and ML analyses were performed in MEGA X (Kumar et al. 2018). Branch support was estimated using 1000 bootstrapping replications. The partitioned BI analysis was performed in MrBayes v. 3.2.7 (Ronquist et al. 2012) with 2,000,000 generations and a sampling frequency of 100 generations. The first 25% of the obtained trees were discarded as burn-in. Although three methods were used to construct the phylogeny, only the BI tree was selected for presentation in this study because the three methods revealed congruent topologies. Phylogenetic trees were arranged and edited using FigTree v. 1.4.0 (Rambaut 2012).

### Abbreviations

**ag**, albumin gland; **at**, atrium; **ep1**, proximal part of epiphallus nearer to penis; **ep2**, distal part of epiphallus nearer to retractor muscle; **fl**, flagellum; **fo**, free oviduct; **hd**, hermaphroditic duct; **p**, penis; **prm**, penial retractor muscle; **pro**, prostate; **gs**, gametolytic sac (= bursa copulatrix); **so**, spermoviduct; **ut**, uterus; **v**, verge; **va**, vagina; **vd**, vas deferens.

## Results

### DNA sequence data

The aligned COI fragments had a length of 655 bp, while the aligned 16S rRNA fragments had a length of 460 bp. The concatenated dataset, therefore, comprised 1115 bp. The level of genetic divergence, as revealed by the uncorrected COI *p*-distance, among Thai *Landouria* taxa varied from 0.048 to 0.192, whereas interspecific distances among species from Thailand, Timor-Leste, China, and Sumatra, Indonesia ranged from 0.048 to 0.284 (Table 2).

**Table 2.** Estimates of COI sequence divergences (uncorrected *p*-distances) of *Landouria* taxa in this study.

No.	Taxon	1	2	3	4	5	6	7	8	9	10	11	12	13	14	15
1	*L. omphalostoma*															
2	*L. rotatoria*	0.255														
3	*L. winteriana*	0.216	0.239													
4	*L. montana*	0.206	0.244	0.195												
5	*L. timorensis*	0.210	0.227	0.199	0.141											
6	*L. strobiloides*	0.160	0.263	0.221	0.212	0.226										
7	*L. circinata*	0.150	0.278	0.238	0.193	0.207	0.105									
8	*L. tuberculata*	0.154	0.262	0.214	0.204	0.228	0.095	0.100								
9	*L. trochomorphoides*	0.163	0.278	0.232	0.218	0.238	0.111	0.107	0.098							
10	*L. chloritoides*	0.137	0.249	0.212	0.191	0.219	0.093	0.098	0.074	0.105						
11	*L. elegans*	0.167	0.268	0.231	0.208	0.215	0.122	0.114	0.076	0.107	0.095					
12	*L. diplogramma*	0.181	0.276	0.233	0.227	0.237	0.144	0.150	0.137	0.156	0.137	0.152				
13	*L. bella*	0.179	0.262	0.219	0.211	0.213	0.158	0.141	0.150	0.173	0.146	0.152	0.154			
14	*L. monodon*	0.184	0.284	0.227	0.233	0.268	0.141	0.163	0.161	0.183	0.152	0.180	0.104	0.177		
15	*L. tumpeesuwanorum* sp. nov.	0.150	0.275	0.218	0.201	0.223	0.102	0.102	0.075	0.111	0.048	0.092	0.143	0.156	0.159	
16	*L. flagellolonga* sp. nov.	0.182	0.280	0.235	0.233	0.261	0.173	0.177	0.179	0.192	0.165	0.178	0.161	0.182	0.180	0.171

A total of 16 sequences (655 bp) of the COI gene fragment had nucleotide frequencies of 0.3002, 0.1073, 0.1556, and 0.4369 for A, C, G, and T, respectively, and there were 265 variable sites and 206 were parsimony informative. A total of 14 sequences (460 bp) of 16S rRNA gene fragments had nucleotide frequencies of 0.4063, 0.1039, 0.1178, and 0.3720 for A, C, G, and T, respectively, and there were 444 variable sites which 413 were parsimony informative. The concatenated dataset (1115 bp) had nucleotide frequencies of 0.3600, 0.1087, 0.1325, and 0.3988 for A, C, G, and T, respectively, and there were 709 variable sites, of which 619 were parsimony informative.

### Molecular phylogeny

**Figure 2.**
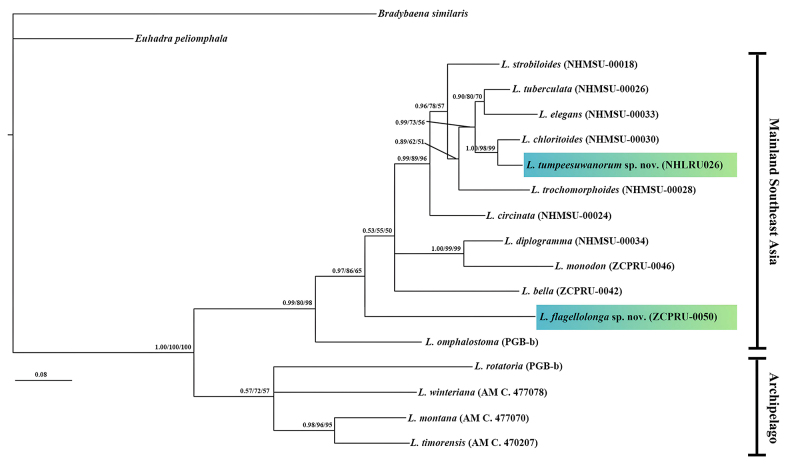
Bayesian inference (BI) tree of the Thai *Landouria* based on concatenated COI and 16S sequences. Numbers at nodes indicate branch support based on posterior probability (BI)/bootstrapping (ML)/bootstrapping (NJ). Scale bar: 0.08 substitutions/site.

The molecular phylogeny was reconstructed using a concatenated dataset of partial COI and 16S rRNA sequences. The trees generated by NJ, ML, and BI analyses were congruent; therefore, only the BI tree is presented (Fig. 2).

The phylogenetic trees divided *Landouria* into two major clades which were strongly supported by a BI posterior probability of 1.00, and 100% for ML and NJ bootstrapping (Fig. 2). The Mainland Southeast Asia clade contains Chinese and Thai taxa (11 species), while the Archipelago clade contains Timor-Leste and Sumatran taxa (4 species)

*Landouria flagellolonga* sp. nov. was clearly separated from congeners with strong support (0.97 for BI posterior probability, and 86% and 65% bootstrap support for ML and NJ, respectively) (Fig. 2). *Landouria flagellolonga* sp. nov. is the sister to the Mainland Southeast Asia clade comprising of all other *Landouria* from Thailand and Chinese with the uncorrected COI *p*-distance within this clade ranged from 0.161 to 0.192 (Table 2).

*Landouria tumpeesuwanorum* sp. nov. is a sister taxon to *L. chloritoides*, with very strong support (1.00 for BI posterior probability, and 98% and 99% bootstrap support for ML and NJ, respectively) (Fig. 2). The uncorrected COI *p*-distance between *L. tumpeesuwanorum* sp. nov. and *L. chloritoides* was 0.048, while *p*-distances among other *Landouria* within this clade ranged from 0.075 to 0.159 (Table 2).

### Systematics

#### Family Camaenidae Pilsbry, 1895

**Subfamily Bradybaeninae Pilsbry, 1934**

**Tribe Aegistini Kuroda & Habe, 1949**

##### Genus Landouria

Taxon classificationAnimaliaStylommatophoraCamaenidae

Godwin-Austen, 1918

DF5ACAC5-30BE-51D5-B92A-E1C5F92A8C3C

###### Type species.

*Helix huttonii* L. Pfeiffer, 1842 (new name for *Helix orbicula* Hutton & Benson, 1838), by original designation.

###### Type locality.

Himalaya near Simla, Mahasu, northern India (Hutton and Benson 1838).

##### Landouria tumpeesuwanorum

Taxon classificationAnimaliaStylommatophoraCamaenidae

Nahok & K. Tanmuangpak sp. nov.

01DF3134-816B-596E-A019-6FE21C399EB2

https://zoobank.org/F27A30E1-4ADF-489C-9876-D69A19C6DAB7

**Figure 3.**
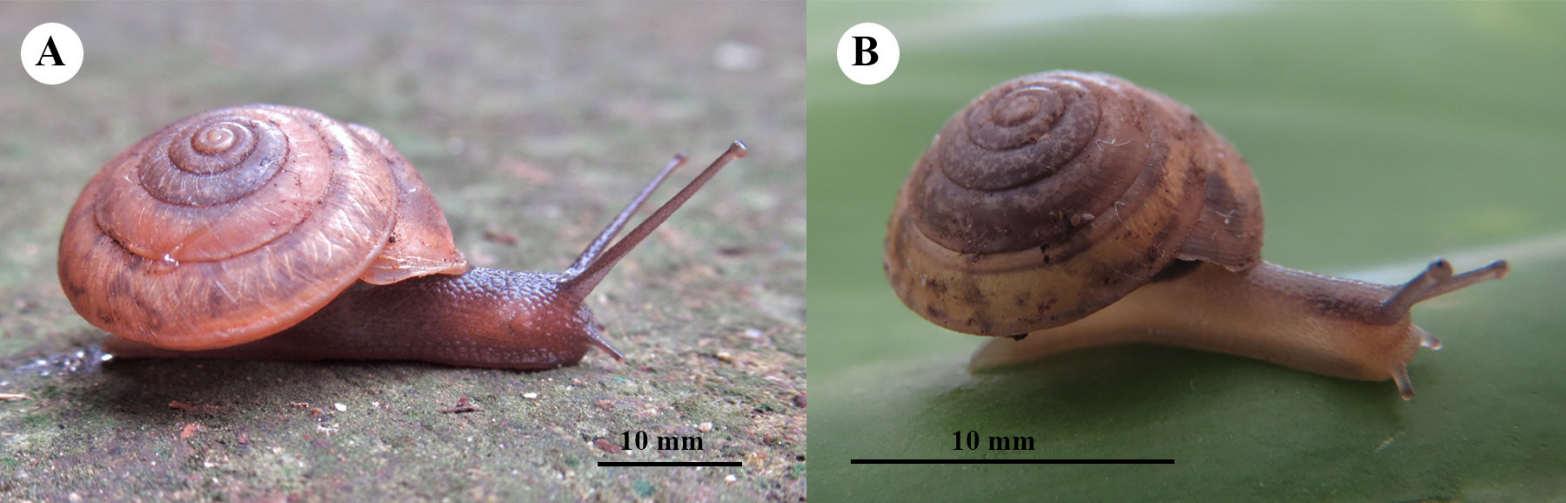
Living adults of two *Landouria* new species. **A**. *L. tumpeesuwanorum* sp. nov. (paratype: NHLRU027); **B**. *L. flagellolonga* sp. nov. (paratype: ZCPRU-0050).

**Figure 4.**
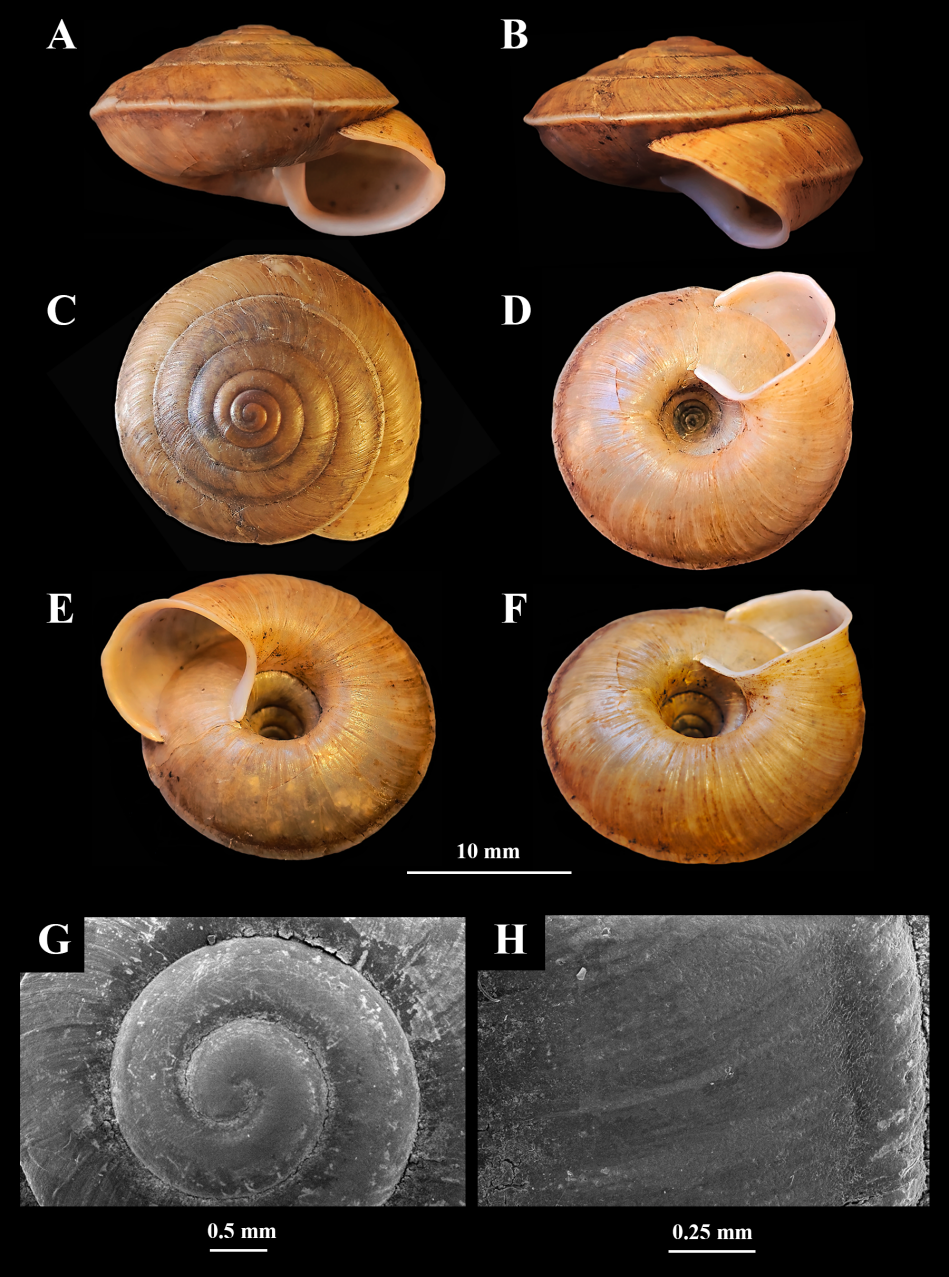
Holotype of *Landouria tumpeesuwanorum* sp. nov. (NHLRU025). **A**. Apertural view; **B**. Lateral view; **C**. Dorsal view; **D**. Ventral view; **E, F**. Umbilical groove; **G**. Protoconch; **H**. Shell surface.

**Figure 5.**
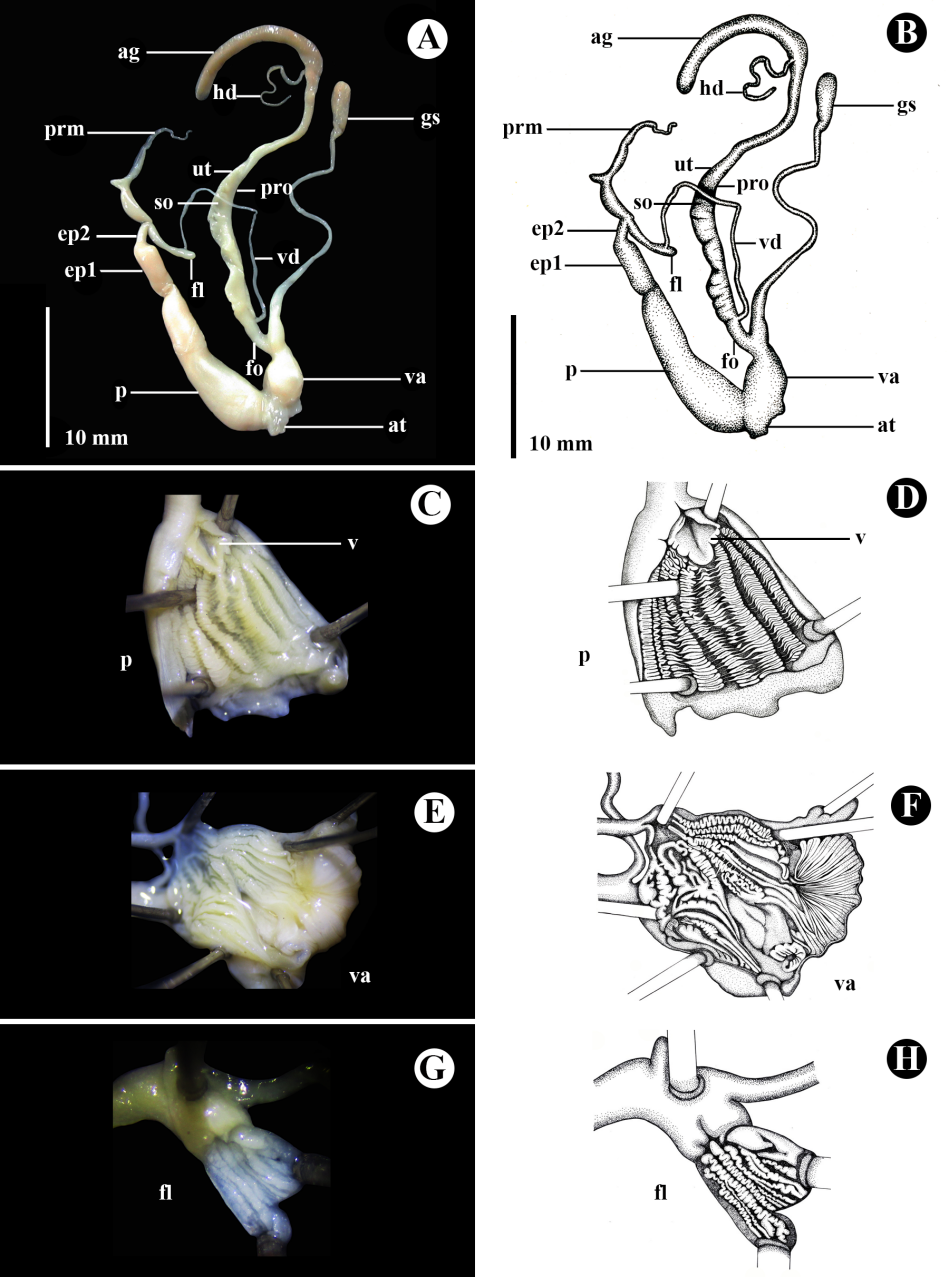
Photograph and schematic drawing of the genital system of *Landouria tumpeesuwanorum* sp. nov., paratype (NHLRU027). **A, B**. Whole genitalia; **C–H**. Internal wall sculpture of **C, D** Penis (p), **E, F** Vagina (va), **G, H** Flagellum (fl). Photograph and drawing by Kitti Tanmuangpak.

**Figure 6.**
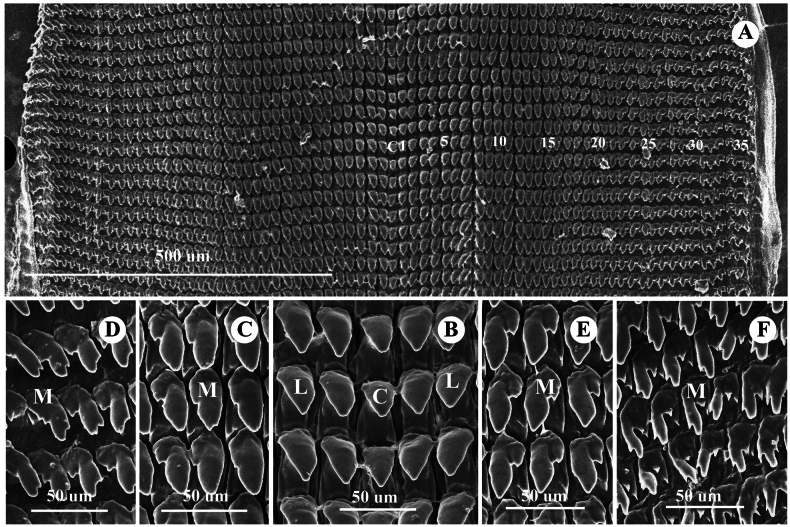
Radula morphology of *Landouria tumpeesuwanorum* sp. nov., paratype (NHLRU027). **A**. Radula plate; **B**. Enlarged view of central tooth and lateral teeth; **C, D**. Enlarged view of left side of marginal teeth; **E, F**. Enlarged view of right side of marginal teeth and central teeth. Numbers indicate the order of lateral and marginal teeth. Abbreviations: C = central tooth, L = lateral teeth, M = marginal teeth.

Figs 3A, 4–6; Table 1

Landouria sp. 1—Tanmuangpak and Kaewsawang 2025: 23; figs 2r, 3n; Table 1.

###### Type locality.

Thailand, Nong Bua Lamphu Province, Si Bun Rueang District, Pha Sam Yod, 17°11'12.01"N, 102°1'54.00"E, limestone hill, alt. 373 m, 24 Nov. 2021, Kitti Tanmuangpak leg.

###### Type specimens.

***Holotype***: NHLRU025 (Fig. 4), SH = 11.24 mm, SW = 18.25 mm. AH = 9.21 mm, AW = 6.41 mm. ***Paratypes***: NHLRU026, 9 shells, NHLRU027 (Fig. 3A), 6 living specimens preserved in ethanol, same collector and locality as holotype.

###### Diagnosis.

Shell large, brownish-corneous, angulated whorls, strongly keeled. Flagellum small with blunt ends; epiphallus cylindrical and abruptly tapering at its distal part; penis swollen at its basal; vagina large and bulged. Radula with triangular central and lateral teeth.

###### Description.

(empty shells = 10, living specimens = 6) ***Shell*** (Fig. 4) dextral, large, depressed-conical, SH = 9.32–12.56 mm (10.75 ± 1.11 mm), SW = 16.02–19.51 mm (17.54 ± 1.15 mm), AH = 8.03–9.54 mm (8.60 ± 0.58) and AW = 4.03–6.41 mm (5.30 ± 0.82), with 6–6.75 slightly convex whorls. Protoconch smooth. Teleoconch radially elongated wrinkles. Apex obtuse and depressed. Suture shallow. Body whorl ridged and with a thick, distinctly downwards bent keel. Umbilicus deep and wide (occupying c. 1/3 of SW). Aperture slightly tilted from axis of coiling, with weakly thickened and weakly expanding lip.

***Genital system*** (*N* = 3) (Fig. 5). Atrium short. Dart apparatus and mucous glands absent. Penis longer than epiphallus, anterior portion a large tube and bulged, and distal end a cylindrical tube and narrowed, internally with nine thick, corrugated longitudinal pilasters; opening of grooved verge (Fig. 5C, D). Epiphallus connected to distal end of penis, proximal part (ep1) thickened at base a cylindrical tube, whereas distal part (ep2) very short and narrow. Penial retractor muscle present and inserted in middle of epiphallus. Flagellum short, cylindrical, small, and blunt apically, internally with 10 corrugated longitudinal pilasters, varying in size (Fig. 5G, H). Vas deferens a long, slender tube, laterally entering epiphallus. Vagina large and stout, longer than free oviduct, internally with 10 corrugated longitudinal pilasters, varying in size (Fig. 5E, F). Free oviduct a short and cylindrical tube. Gametolytic sac a long, cylindrical tube, anterior portion bulging and connected to free oviduct, distal end a slender tube, and with small, oval sac at distal end. Spermoviduct longer than vagina; uterus large, with very thin prostate gland adhering to it.

***Radula*** (*N* = 3) (Fig. 6). Comprises 118–120 transverse rows with 75–76 teeth per row, formula: (23–25)+(13–15)+1+(13–15)+(23–25). Central tooth usually symmetric, unicuspid, triangular (Fig. 6B). Lateral teeth unicuspid, longer and larger than central teeth (Fig. 6B). Marginal teeth asymmetrical, gradually changing from bicuspid to finally tricuspid; endocone small; mesocone large, with curved margins; ectocone triangular and located at tooth base (Fig. 6C–F).

###### Etymology.

In honour of Assoc. Prof. Chanidaporn Tumpeesuwan and Sakboworn Tumpeesuwan, Thai malacologists and our beloved advisor and co-advisor, respectively, who initiated the taxonomic study of *Landouria* in Thailand and whose inspiration has led to our study of molluscs.

###### Habitat.

This new species lives in limestone hill areas in mixed-deciduous forest.

###### Distribution.

*Landouria tumpeesuwanorum* sp. nov. is currently known from limestone hills at the boundary between Loei Province and Nong Bua Lamphu Province, northeastern Thailand (Fig. 1).

###### Remarks.

The shell morphology of the new species is similar to *L. strobiloides* (see Tumpeesuwan and Tumpeesuwan 2019: fig. 3) and *L. circinata* (see Nahok et al. 2021: fig. 3A). The shell of *L. tumpeesuwanorum* sp. nov. is larger, with a thicker keel at the periphery, and consistently brownish-corneous. *Landouria strobiloides*, however, is characterised by having pale-brown early whorls gradually changing to dark brown on the body whorl, while *L. circinata* is light brown on the first four whorls and dark brown on the last two whorls.

The genital anatomy of *L. tumpeesuwanorum* sp. nov. is quite similar to that of *L. chloritoides*. The new species slightly differs from *L. chloritoides* in that its epiphallus is divided into two parts: the proximal part (ep1) is thick, whereas the distal part (ep2) is shorter, smaller, and bears a small flagellum with a blunt apex. In contrast, *L. chloritoides* has a thick epiphallus that is not clearly divided into two parts (ep2 is very short), and the flagellum is attached to the penial retractor muscle and is elongate-ovate. Regarding shell morphology, *L. chloritoides* has a stout body whorl that is usually made slightly angular by a blunt peripheral keel, whereas *L. tumpeesuwanorum* sp. nov. has angulated whorls with a strong keel.

##### Landouria flagellolonga

Taxon classificationAnimaliaStylommatophoraCamaenidae

 Nahok & K. Tanmuangpak sp. nov.

A542E450-A6C2-5D92-AD87-65ACFBDB40F8

https://zoobank.org/E599C38C-A980-433A-B8A6-CD3ACA28A706

**Figure 7.**
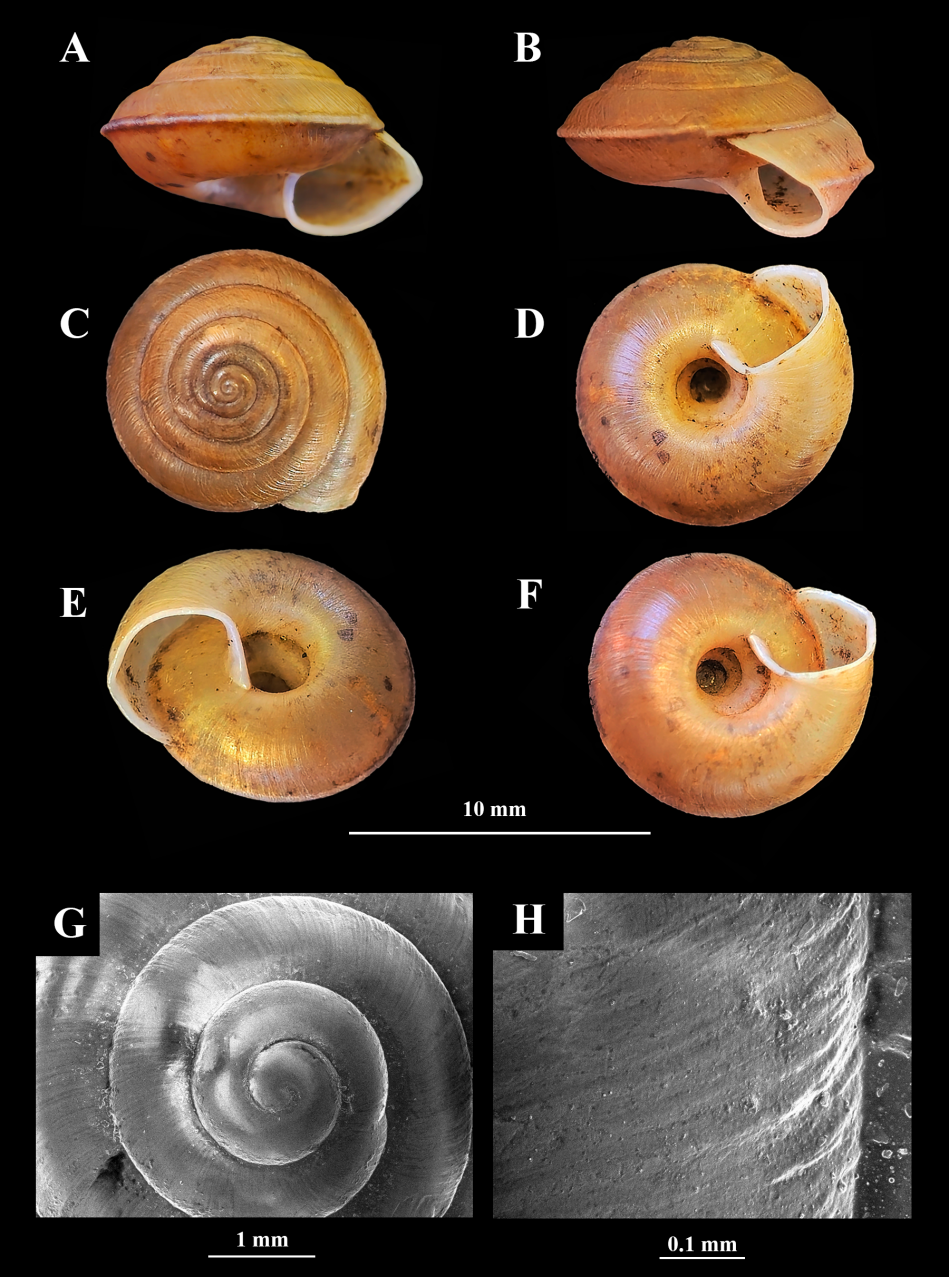
Holotype of *Landouria flagellolonga* sp. nov. (ZCPRU-0048). **A**. Apertural view; **B**. Lateral view; **C**. Dorsal view; **D**. Ventral view; **E, F**. Umbilical groove; **G**. Protoconch; **H**. Shell surface.

**Figure 8.**
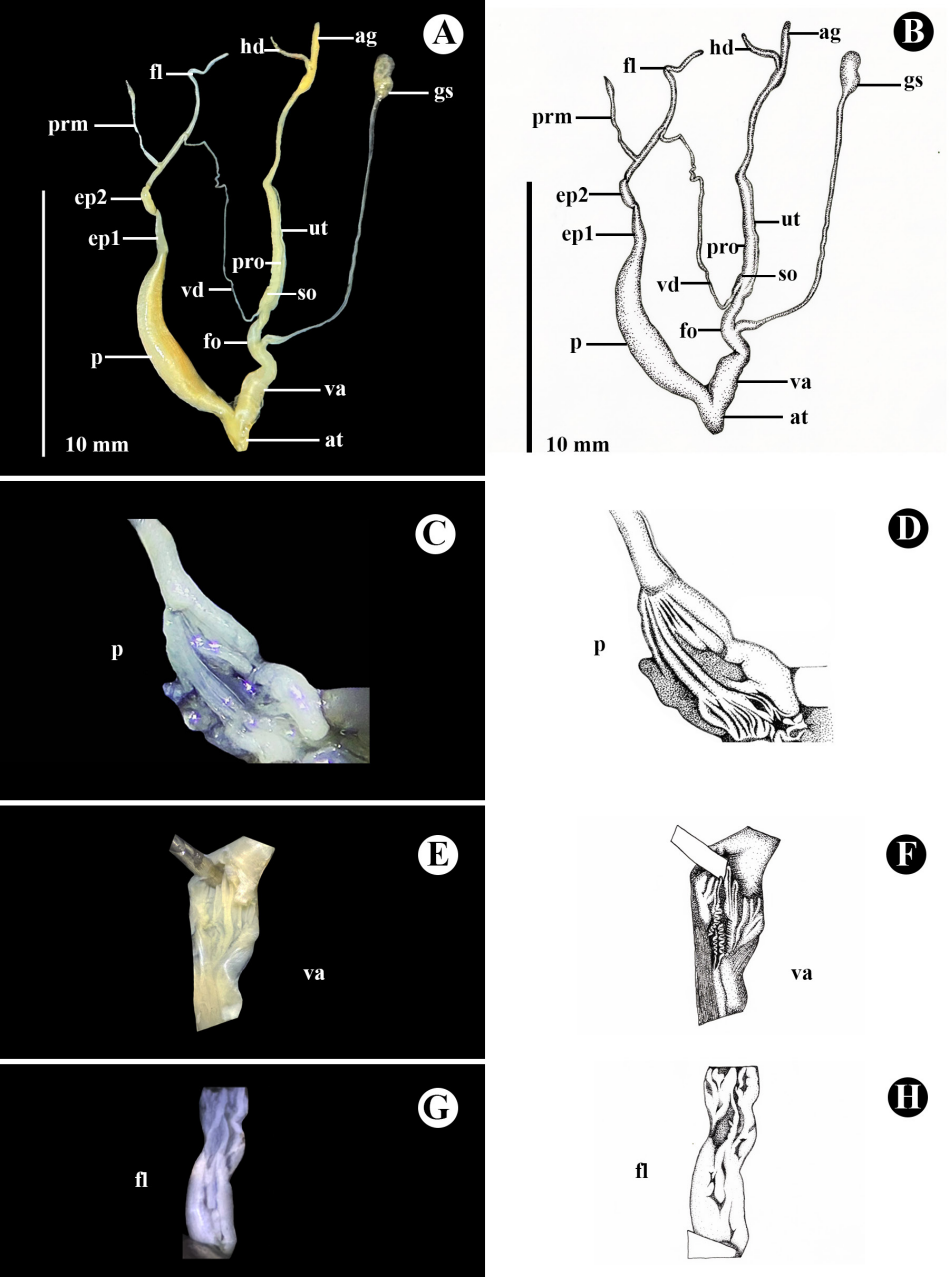
Photograph and schematic drawing of the genital system of *Landouria flagellolonga* sp. nov., paratype (ZCPRU-0050). **A, B**. Whole genitalia; **C–H**. Internal wall sculpture of **C, D** Penis (p), **E, F** Vagina (va), **G, H**. Flagellum (fl). Photograph by Benchawan Nahok, drawing by Kitti Tanmuangpak.

**Figure 9.**
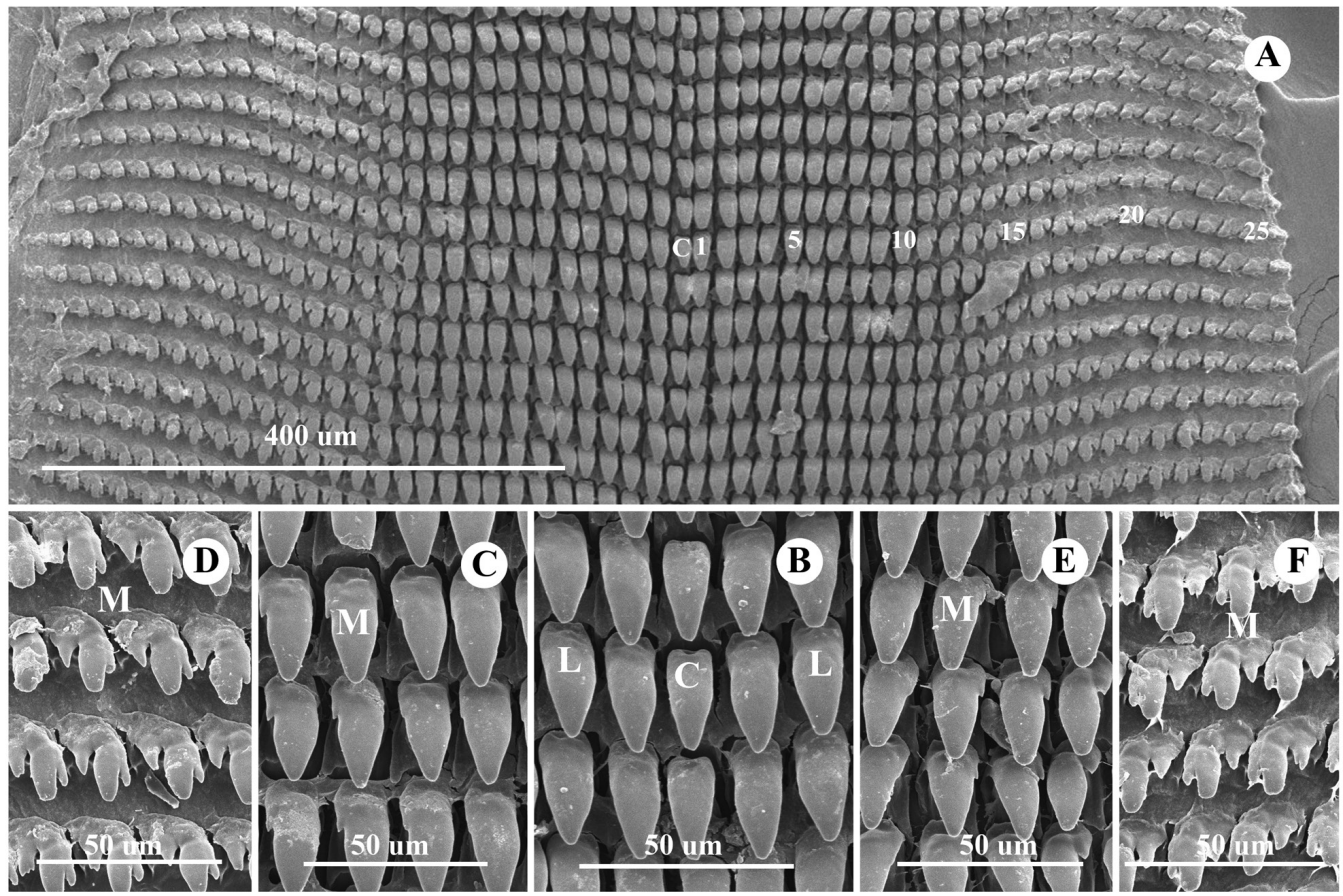
Radula morphology of *Landouria flagellolonga* sp. nov., paratype (ZCPRU-0050). **A**. Radula plate; **B**. Enlarged view of central tooth and lateral teeth; **C, D**. Enlarged view of left side of marginal teeth; **E, F**. Enlarged view of right side of marginal teeth and central teeth. Numbers indicate the order of lateral and marginal teeth. Abbreviations: C = central tooth, L = lateral teeth, M = marginal teeth.

Figs 3B, 7–9; Table 2

Landouria sp. 10—Nahok 2020: 55; figs 24A, 33; table 3.

###### Type locality.

Thailand, Sa Kaeo Province, Khlong Hat District, Phet Pho Thong Cave, 13°24'52.89"N, 102°19'31.03"E, limestone hill, alt. 236 m, 15 Oct. 2017, Benchawan Nahok and Utain Chanlabut leg.

###### Type specimens.

***Holotype***: ZCPRU-0048 (Fig. 7), SH = 6.80 mm, SW = 9.65 mm. AH = 4.72 mm, AW = 3.60 mm. ***Paratypes***: ZCPRU-0049 30 shells, ZCPRU-0050 (Fig. 3B), 12 living specimens preserved in ethanol, same collector and locality as holotype, 27 Aug. 2025.

###### Diagnosis.

Shell small, light brown-corneous, sharply keeled. Flagellum very long, epiphallus long and slender; penis long, cylindrical, dilated at its middle part; vagina and free oviduct short. Radula with lanceolate central and lateral teeth.

###### Description.

(empty shells = 31, living specimens = 12) ***Shell*** (Fig. 7) dextral, small, conical-lenticular, SH = 5.90 –7.50 mm (6.70 ± 0.46 mm), SW = 8.20–11.10 mm (9.65 ± 0.84 mm), AH = 3.50–5.30 mm (4.40 ± 0.52 mm), and AW = 3.50–5.00 mm (4.25 ± 0.43 mm), with 6–6.25 slightly convex whorls. Protoconch almost smooth. Teleoconch with irregular wrinkles; incised spiral lines not discernible. Apex obtuse and depressed. Suture shallow. Body whorl sharply keeled. Umbilicus open, relatively wide (occupying c. 1/3 of SW). Aperture rounded rhombic; apertural lip reflexed and thickened.

***Genital system*** (*N* = 3) (Fig. 8). Atrium short. Dart apparatus and mucous glands absent. Penis twice as long as epiphallus, long, cylindrical, broad in middle and narrows at distal end, internally with five narrow longitudinal pilasters, without a verge (Fig. 8C, D). Epiphallus divided into two somewhat identical parts; proximal part (ep1) connected to penis, slightly larger and longer than distal part (ep2). Penial retractor muscle present. Flagellum very long, without node, tapering towards its distal end, internally with a few fine longitudinal folds (Fig. 8G, H). Vas deferens thin and long. Vagina approximately 1/2 of penis length, internally with six smooth, longitudinal pilasters (Fig. 8E, F); free oviduct short, regularly cylindrical tube. Gametolytic sac slightly thicker at base, with long, narrow, thin cylindrical tube, and at its distal end, a small, swollen spherical sac. Prostate gland very long. Uterus long and thin.

***Radula*** (*N* = 3) (Fig. 9). Comprises 121–125 transverse rows with 53–61 teeth per row, formula: (17–19)+(9–11)+1+(9–11)+(17–19). Central tooth usually symmetric, unicuspid, lanceolate (Fig. 9B). Lateral teeth unicuspid, elongate-lanceolate, oblique, and longer than central teeth (Fig. 9B). Marginal teeth gradually changing from unicuspid to bicuspid and finally tricuspid (Fig. 9C–F).

###### Etymology.

Specific epithet derived from Latin word *longus*, meaning “long” and referring to the elongated flagellum of this species.

###### Habitat.

This species inhabits limestone hills with scattered outcrops, bare ground, and trees in a natural forest.

###### Distribution.

*Landouria flagellolonga* sp. nov. is known from the surroundings of limestone hills at Phet Pho Thong Cave, Khlong Hat District, Sa Kaeo Province, eastern Thailand (Fig. 1).

###### Remarks.

*Landouria flagellolonga* sp. nov. differs from other Thai species of *Landouria* by its genital system, which has the most slender and longest flagellum (Fig. 8).

## Discussion

*Landouria tumpeesuwanorum* sp. nov. is described from northeastern Thailand, while *L. flagellolonga* sp. nov. is described from eastern Thailand with the first anatomically verified record. Most Thai *Landouria* species exhibit allopatric distributions. Although some species occur in the same province, their geographic ranges do not overlap, as they are separated by geographical barriers and are typically endemic to small areas within the limestone hills of Thailand.

All members of Thai *Landouria* share the character combination of the absence of a dart apparatus and mucous glands (Tumpeesuwan and Tumpeesuwan 2019; Nahok et al. 2021, 2024) and a depressed shell, often with an angulated periphery or keel, and with a wide, open umbilicus. These are common conchological features of the genus *Landouria* (Schileyko 2004; Köhler et al. 2018; Nurinsiyah et al. 2019).

Our investigation, which considered both genitalia and DNA sequences, recognized 11 species of *Landouria* from Thailand based on the available material and presented two new species. The interspecific COI sequence divergences between Thai *Landouria* species were high (4.80–19.20%). The most closely related species are *L. tumpeesuwanorum* sp. nov. and *L. chloritoides*. In contrast, the most distantly related are *L. flagellolonga* sp. nov. and *L. trochomorphoides*. Further, the substantial genetic distances between these morphologically delimited species from Thailand (Table 2) support the conclusion that these populations truly represent distinct species.

### Key to Thai *Landouria* species by shell morphology

**Table:** 

1	Shell without peripheral keel	**2**
–	Shell with peripheral keel	**3**
2	Shell depressed-globose, without brown band	***L. bella***
–	Shell low-conical, with brown band	***L. diplogramma***
3	Inner side of aperture present columellar lamella	**4**
–	Inner side of aperture without columellar lamella	**5**
4	Columellar lamella present blunt tooth at the basal	***L. canalifera***
–	Columellar lamella without blunt tooth at the basal	***L. monodon***
5	Peripheral keel blunt	***L. chloritoides***
–	Peripheral keel sharp	**6**
6	Peripheral keel slightly sharp, shell surface with numerous tiny tubercles	***L. tuberculata***
–	Peripheral keel very sharp, shell surface without tubercles	**7**
7	Shell with low spire, shell surface with radial scaly processes	***L. trochomorphoides***
–	Shell with high spire, shell surface without radial scaly processes	**8**
8	Keel with downward bent rim	***L. elegans***
–	Keel without downward bent rim	**9**
9	Suture indented, growth line obvious	***L. strobiloides***
–	Suture not indented, growth line obscure	**10**
10	Peripheral colour band present	***L. circinata***
–	Peripheral colour band absent	**11**
11	whorl keeled with keel edge distinctly bent downwards	***L. tumpeesuwanorum* sp. nov**.
–	Body whorl keeled but keel edge not bent downwards	***L. flagellolonga* sp. nov**.

### Key to Thai *Landouria* species by genital characters

**Table:** 

1	Penis shorter than vagina; flagellum strobilus-like	***L. strobiloides***
–	Penis longer than vagina; flagellum non-strobilus-like	**2**
2	Flagellum circinate	***L. circinata***
–	Flagellum with a short protrusion, either ovate and slender, or long and cylindrical	**3**
3	Tip of flagellum blunt	***L. tumpeesuwanorum* sp. nov**.
–	Tip of flagellum not blunt	**4**
4	Inner sculpture of penis with parallel, transverse folds	***L. trochomorphoides***
–	Inner wall of penis with longitudinal pilasters	**5**
5.	Inner wall of flagellum with sparse, longitudinal pilasters	***L. flagellolonga* sp. nov**.
–	Inner sculpture of flagellum with dense, longitudinal pilasters	**6**
6	Rounded verge absent	***L. elegans***
–	Rounded verge present	**7**
7	Epiphallus clearly divided into two portions (ep1 and ep2)	***L. diplogramma***
–	Epiphallus not clearly divided into two portions (ep2 is very short)	**8**
8	Vagina as long as free oviduct	***L. chloritoides***
–	Vagina shorter than free oviduct	**9**
9	Basal part of gametolytic sac slightly swollen	***L. tuberculata***
–	Basal part of gametolytic sac more swollen	**10**
10	Inner wall of vagina with thick longitudinal pilasters	***L. bella***
–	Inner wall of vagina with thick undulating transverse pilasters	***L. monodon***

## Supplementary Material

XML Treatment for Landouria

XML Treatment for Landouria tumpeesuwanorum

XML Treatment for Landouria flagellolonga

## Additional information

### Artificial Intelligence (AI) use

The authors accept full responsibility for the content of the manuscript, including the disclosure of any use of AI.

No AI tools were used in the preparation of this manuscript.

### Author ORCIDs

B. Nahok https://orcid.org/0009-0009-1788-6788

U. Chanlabut https://orcid.org/0000-0001-8275-7434

K. Tanmuangpak https://orcid.org/0000-0003-2968-3506

### Data availability

All of the data that support the findings of this study are available in the main text.
